# PFKFB3: A Potential Key to Ocular Angiogenesis

**DOI:** 10.3389/fcell.2021.628317

**Published:** 2021-03-11

**Authors:** Zi-Yi Zhou, Lin Wang, Yu-Sheng Wang, Guo-Rui Dou

**Affiliations:** ^1^Department of Ophthalmology, Xijing Hospital, Eye Institute of Chinese PLA, Fourth Military Medical University, Xi’an, China; ^2^Department of Hepatobiliary Surgery, Xijing Hospital, Fourth Military Medical University, Xi’an, China

**Keywords:** ocular angiogenesis, PFKFB3, endothelial metabolism, glycolysis, endothelial cell

## Abstract

The current treatment for ocular pathological angiogenesis mainly focuses on anti-VEGF signals. This treatment has been confirmed as effective despite the unfavorable side effects and unsatisfactory efficiency. Recently, endothelial cell metabolism, especially glycolysis, has been attracting attention as a potential treatment by an increasing number of researchers. Emerging evidence has shown that regulation of endothelial glycolysis can influence vessel sprouting. This new evidence has raised the potential for novel treatment targets that have been overlooked for a long time. In this review, we discuss the process of endothelial glycolysis as a promising target and consider regulation of the enzyme 6-phosphofructo-2-kinase/fructose-2,6-bisphosphatase as treatment for ocular pathological angiogenesis.

## Introduction

Ocular vasculature formation is characterized by a process of neovascularization, vessel maturation, and degeneration, which is orchestrated by a series of complex and precise mechanisms. Lined by quiescent endothelial cells (ECs), the retinal vasculature and choroidal vasculature supply oxygen and nutrients to every cell in the visual system, which supports homeostasis in the eye. However, slight changes in the microenvironment may disrupt the static state and arouse pathological vascular growth, resulting in ocular neovascular diseases.

ECs, as effector cells, play a critical role in the process of angiogenesis. As a response to a stimulus, such as vascular endothelial growth factor (VEGF), quiescent ECs become active and phenotypic alterations to migratory tip cells or proliferative stalk cells, initiating the angiogenic process. Many studies in the past century have aimed to block the angiogenetic functions of ECs and their results have been the basis for remarkable advances (Carmeliet and Jain, 2011; Potente et al., 2011). For instance, anti-VEGF therapy has been widely applied in angiogenic ocular diseases. However, as VEGF is also a neurotrophic factor and cardiovascular protective factor, VEGF deprivation is associated with some inevitable problems.

In recent decades, endothelial metabolism has become a research target (Jacobs and Gavard, 2018; Li and Carmeliet, 2018; Li et al., 2019b). To function normally in both quiescent and angiogenic stages, ECs require energy that is produced in several metabolic pathways. The critical role of glycolysis in ECs is prominent because it provides most of the energy that is required for angiogenesis. Similar to reducing the fuel for an overheated metabolic (glycolytic) engine in angiogenic ECs, inhibiting the process of glycolysis may attenuate pathological ocular angiogenesis and may leave fewer systemic side effects than current conventional treatments (Fraisl et al., 2009). Recently, a few encouraging studies have suggested targeting EC glycolysis as a potential alternative to anti-angiogenic therapy. Accordingly, in this review, we discuss glycolysis in ECs and the regulating enzyme 6-phosphofructo-2-kinase/fructose-2,6-bisphosphatase (PFKFB) according to recent studies that aimed to raise alternative strategies for the treatment of neovascular diseases, especially for ocular neovascular diseases.

## Cellular Aspects in the Process of Ocular Angiogenesis

Retinal vasculature formation undergoes a process of remarkable reorganization and changes during development. In brief, blood enters the eye, switching from arriving through the hyaloid vasculature in the early stage of gestation to arriving through the retinal vasculature in mid-gestation. It has been suggested that the switch is triggered by the invasion of astrocytes into the retina (Stone and Dreher, 1987), which is initiated from the optic nerve (Saxena and Meyer, 2015). There are two different mechanisms in human retinal vascularization: vasculogenesis and angiogenesis (Aiello et al., 1994). Vasculogenesis is the re-formation of blood vessels through the aggregation of endothelial precursors. Blood vessels develop from vascular precursor cells (VPCs), which aggregate into solid vascular cords. They then become unobstructed and differentiate into primitive endothelial tubes. On the other hand, angiogenesis occurs with budding from existing blood vessels, which is subsequently realized by migration and proliferation of vascular ECs (Chan-Ling et al., 1990). The original blood vessels formed by angiogenesis are not capable of meeting the metabolic needs of the posterior retina because the capillaries remain as small branches. The density of capillaries in the posterior part of the retina expands from the original blood vessels by means of angiogenesis. During the development of the retina, the increase in metabolic demands of activated neurons cause local “physiological hypoxia,” which triggers vascular growth in the area.

Retinal angiogenesis is a highly regulated and complex process. We can clearly observe the process of angiogenesis during the elongation of the primary plexus. At the very beginning, the vessels spread from vascular areas to avascular areas in the form of short sprouts. They then fuse with other short sprouts in the area, which may have potential relations to the microglial in the retina (Checchin et al., 2006). The factors contributing to retinal angiogenesis are fairly complex. VEGF is one of the critical candidates and it is stimulated by the hypoxia environment that the sprouts confronted during the process of angiogenesis in the avascular areas (Stone et al., 1995). VEGF regulates the migration and proliferation of vascular endothelial cells. The distribution of VEGF from the center to the edge of retinal vessels has a concentration gradient that can regulate the growth rate and directions of retinal vessels (Gerhardt et al., 2003). The newly formed immature capillaries are intended to degenerate, while the more mature and larger blood vessels can resist various harmful effects from the external environment. Thus, after the formation of primary loops and networks, profound remodeling takes place to build a more mature, functional, and structured vessel tree. The process of remodeling includes vascular degeneration and vascular stabilization. Blood perfusion (Chen et al., 2012), endothelial cell apoptosis (Franco et al., 2015), and endothelial cell migration (Franco et al., 2015) contribute to the process. The retinal vasculature develops and the hyaloid vasculature degenerates completely by 38–40 WG (Gariano, 2003). Unlike vascularization in humans, rodent retinal vascularization occurs after birth (Fruttiger, 2007).

## The Ocular Pathological Angiogenic Process

Pathological angiogenesis is characterized by leaky and immature vessels and the development of pathological neovascularization may gradually lead to retinal detachment and visual damage (Al-Latayfeh et al., 2012). The presence of pathological angiogenesis can be observed in many ocular diseases, such as retinopathy of prematurity (ROP), proliferative diabetic retinopathy (PDR), and age-related macular degeneration (AMD).

Take ROP as an example. As was first reported in 1942 (Terry, 2018), ROP has been a continuously leading cause of blindness in premature infants worldwide, especially in middle-income countries (Gilbert et al., 2005). Globally, in 2010, an estimated 184,700 babies of 14.9 million preterm babies developed any stage of ROP, and 20,000 of them become blind (visual acuity <20/400) or severely visually impaired (visual acuity from <20/200 to ≥20/400) due to ROP (Blencowe et al., 2013; Kim et al., 2018). Development of the retinal vasculature begins at 16 WG (Roth, 1977) and is completed by around 36 WG–40 WG (Roth, 1977). This implies that, when born preterm, the retinal vessels are not mature at birth. An oxygen-induced retinopathy (OIR) model in mice has become a classic model that partially mimics the pathological angiogenic process in ROP (Smith et al., 1994). To build a pathological model, the mice on postnatal day 7 (P7) were placed in a sealed oxygen box with the oxygen concentration maintained at 75 ± 2%; after five days of lactation, the mice and their lactating mothers on postnatal day 12 (P12) were put back into the indoor standard environment (where the concentration of oxygen was 21%) (Smith et al., 1994). Exposure to the relatively high concentration of oxygen outside the uterus with continuous oxygen delivery, compared to inside the uterus, leads to the first phase of the pathological process, which is characterized by vessel regression. The arterial oxygen partial pressure is much higher than the intrauterine pressure, which causes the retina to fill with oxygen from the choroidal vascular system with poor auto-regulation (Kur et al., 2012), thus offsetting the physiological hypoxia stimulation required for normal retinal vascularization. At this time, only partial vascularization has been observed in the developing retina. Hyperoxia leads to the downregulation of VEGF expression in astrocytes, Müller cells (Stone et al., 1995), pericytes (Darland et al., 2003), and the RPE through the hypoxia inducible factor alpha (HIF-1a) (Shima et al., 1995). The loss of vascular endothelial cell proliferation and survival factors leads to apoptosis and vascular degeneration, together with the cessation of angiogenesis (Chan-Ling et al., 1992; Alon et al., 1995). Therefore, during the period of neonatal hyperoxia exposure, the rate of angiogenesis slows down while the maturation of neural elements remains unchanged (Tailoi et al., 1995; Hartnett and Penn, 2012). This results in an avascular retinal area with active metabolism, which provides a hypoxia stimulus for pathological neovascularization in ROP (Tailoi et al., 1995). The second phase that follows is promoted by the hypoxia caused by the absence of a vasculature that conveys abundant oxygen and nutrients. At this stage, the metabolic needs of retinal development are no longer met by an exogenous oxygen supply. Due to the delayed vascularization process related to neuronal maturation, retinal hypoxia leads to the upregulation of VEGF expression and the recovery of vascular growth. Sensing the low concentration of oxygen, the expression of HIF-1a is upgraded to initiate transcription of the genes responding to hypoxia, such as *VEGF*. Thus, pathological angiogenesis begins (Hoppe et al., 2016). Moreover, a study has shown that expression of the adenosine A2a receptor (ADORA2A) is markedly increased in the retina of mice with OIR, which promotes hypoxia-inducible transcription factor-1(HIF-1)-dependent endothelial cell glycolysis and is crucial for pathological angiogenesis (Liu et al., 2017). Pathological vessels in the second stage of ROP grow with a significant increase of VEGF, when it is absent with astrocyte encapsulation and an intact blood-retinal barrier (BRB) (Chan-Ling et al., 1992). The leaky and non-functional vessels may finally grow into the vitreous, resulting in detachment of the retina and irreversible visual damage if left untreated.

Considering the fundamental role of VEGF in angiogenesis, anti-VEGF therapy has become increasingly popular in the treatment of ocular angiogenesis. In fact, anti-VEGF therapy has been widely used in current clinical applications, including ROP, wet AMD and PDR (Alon et al., 1995; Mintz-Hittner and Kuffel, 2008; Penn et al., 2008; Mintz-Hittner et al., 2011; Carroll and Owen, 2020). However, concerns still exist on the potential local and systemic side effects of intraocular anti-VEGF therapy (Geloneck et al., 2014; Wu and Wu, 2018; Natarajan et al., 2019; Raghuram et al., 2019). As is commonly acknowledged, VEGF is generally expressed in a variety of cell types in the eye and acts as a neurotrophic factor. Intraocular injection of an inhibitor of VEGF may interrupt ocular development and vasculature reconstruction. Furthermore, the potential leakage of anti-VEGF drugs into the circulation may produce systemic side effects (Carroll and Owen, 2020).

## The Role of Endothelial Cells and Endothelial Cell Metabolism in Angiogenesis

ECs act as a natural barrier between the blood stream and the vascular wall by forming a thin line in the vessels (Cahill and Redmond, 2016). Intriguingly, unlike other healthy cell types, despite access to the oxygen conveyed by blood cells flowing in vessels, ECs prefer the glycolytic pathway over the oxidation of glucose, which is similar to tumor cells. As evidence has indicated, the proliferative ECs generate up to 85% ATP by glycolysis (De Bock et al., 2013b; Bierhansl et al., 2017). This may be explained by the biological function of the vasculature to transfer oxygen and other nutrients to distant tissues. Also, protecting ECs from the accumulated damage of reactive oxygen species (ROS) may be a self-protection mechanism for the body (De Bock et al., 2013a). As ECs switch to an angiogenetic subtype, tip cells migrate to a hypoxia microenvironment in which they can barely utilize the oxidative metabolism. In addition, the generation of ATP is faster by glycolysis, which enables rapid neovascularization in avascular areas (De Bock et al., 2013b).

ECs are considered key players in angiogenesis and they stay quiescent during most developmental periods. Under the circumstances of inflammation or ischemia, ECs switch and enter a proliferative mode, which initiates the process of angiogenesis (Potente and Carmeliet, 2017). The active ECs differentiate into tip cells to lead the migration of vessel sprouts and stalk cells to elongate the stalk of the sprouts. With the filopodia located in the front of the vessel branches, the endothelial tip cells are fairly polarized and are sensitive to the angiogenetic clues in the microenvironment, such as VEGF, so that they are capable of leading the process of angiogenesis (Gerhardt et al., 2003; Tammela et al., 2008). Stalk cells, the other subtype of ECs, are highly proliferative and have the ability to elongate the vessel stalk as well as form the vessel lumen (Ruhrberg et al., 2002; Gerhardt et al., 2003; De Smet et al., 2009). In addition, stalk cells maintain the stability and integrity of neovascularization by forming adhesions and cell-to-cell tight junctions (Jacobs and Gavard, 2018). There is a third subtype of ECs, namely phalanx cells. Once the blood flow is established and the vascular wall is stabilized, ECs return to a quiescent mode and then sense the changes in the microenvironment (De Bock et al., 2009).

In this whole process, ECs adapt their metabolism to proliferate or migrate, and they maintain the vascular barrier and protect themselves against oxidative stress in the high-oxygen environment they are exposed to in healthy conditions at the same time (Dumas et al., 2020). Because of the different phenotypes of ECs at different stages and their different functions, the metabolic pathways and metabolites of ECs with different phenotypes are not the same. In phalanx cells, glycolysis plays a major role in maintaining the basic energy needs of cells (Wilhelm et al., 2016; Kalucka et al., 2018). In tip cells, glycolysis may remodel actin to meet the needs of cell migration (Real-Hohn et al., 2010; Rohlenova et al., 2020), and in stalk cells, it mainly provides energy for cell proliferation (Schoors et al., 2014; Rohlenova et al., 2020).

The metabolic transcriptome of vascular ECs reprograms the process of pathological angiogenesis. When attention is paid to metabolic genes and metabolic pathways in the process of EC differentiation from quiescent veins to angiogenic ECs, it has been observed that the membrane transports ATP synthase, and glycolytic gene signatures are dynamically regulated (Rohlenova et al., 2020). Large differences between immature endothelial cells and tip cells can be observed in the activities of metabolism genes, indicating that highly functioning ECs have higher metabolic demands because of their physiological functions. Solid evidence has confirmed that the glycolytic transcriptome in tumor vessels and vascular endothelial cells in pathological neovascularization is upregulated. Given the influences of the microenvironment, it is understandable that the glycolytic gene signatures are much stronger in tumor ECs than in pathological neovascular diseases (Lyssiotis and Kimmelman, 2017). Furthermore, the deletion of glycolytic genes in ECs or the application of PFKFB3 inhibitors can indeed reduce the proliferation activity of tumor ECs (Cantelmo et al., 2016). This also indicates why targeting endothelial glycolysis is a potential therapy for ocular neovascularization.

Targeting the endothelial metabolism in pathological angiogenesis has become of interest in recent decades. Metabolic pathways also include fatty acid oxidation and amino acid metabolism, while glycolysis of ECs plays a decisive role in the formation of blood vessels. ECs are responsible for the stabilization of stem cell proliferation and vascular regeneration. In recent years, an increasing number of studies have shown that the metabolic disorders of endothelial cells can lead to endothelial dysfunction and vascular disease (Li et al., 2019a). Moreover, as the tip-stalk mechanism plays a significant role in angiogenesis, it is also worth mentioning that the major glycolysis regulator PFKFB3 can equally regulate the differentiation of tip/stalk cells (De Bock et al., 2013b) along with complex regulatory factors, including VEGF (Jin et al., 2017), the Notch/Delta-like 4 (Dll4) signaling pathway (Lobov and Mikhailova, 2018), and FOXO1 (Kim et al., 2019). This is discussed further in a later section.

## Glycolysis in EC Metabolism

**FIGURE 1:**
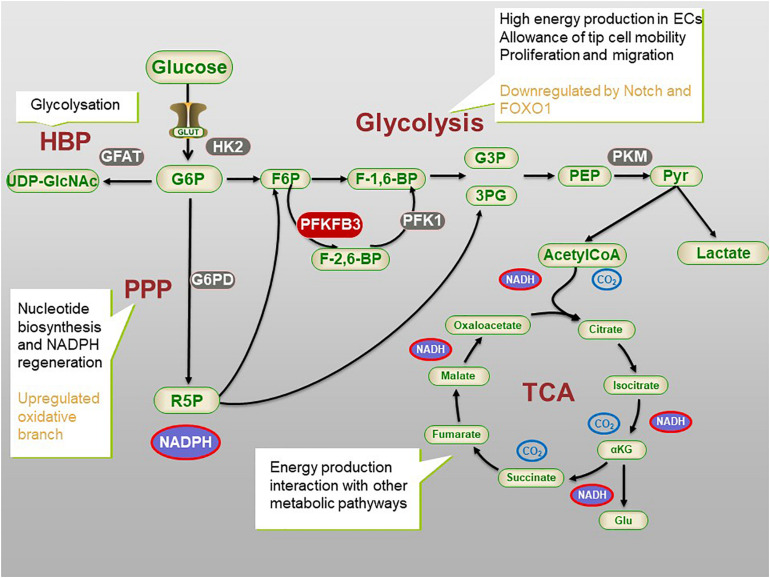
The process of glycometabolism in ECs. The whole process of glycometabolism contains two distinct processes, namely glycolysis and tricarboxylic acid (TCA) cycle, and several pathways. For simplification, not all the metabolites or enzymes are presented. ECs main rely on glycolysis to produce energy and produce nucleotide and NADPH via Pentose phosphate pathway (PPP). Hexosamine biosynthesis pathway (HBP) is related to post-transitional modification. 3PG, 3-phosphogylcerate; G6P, glucose 6-phosphate; F6P, fructose 6-phosphate; F-1,6BP, fructose-1,6-bisphosphate; F-2,6-BP, fructose-2,6-bisphosphate; G3P, glyceraldehyde 3-phosphate; 3PG, 3-phosphoglucerate; G6PD, G6P dehydrogenase; GLUT, glucose transporter; HBP, hexosamine biosynthetic pathway; αKG, α-ketoglutarate; PFK1, phosphofructokinase 1; PFKFB3, 6-phosphofructo-2-kinase/fructose-2,6-bisphosphatase 3; PEP, Phosphoenolpyruvate; Pyr, pyruvate; R-5-P, ribose 5-phosphate; TCA, tricarboxylic acid cycle; UDP-GlcNAc, uridine diphosphate N-acetylglucosamine.

Glucose is utilized by ECs via membrane-bound glucose transporters (GLUTs), thus initiating glycolysis (Uldry and Thorens, 2004). The phosphorylation of glucose to glucose-6-phosphate (G-6-P) is the first step in glycolysis. Hexokinase 2 (HK2), which regulates this step, is one of the rate-controlling enzymes (Yu et al., 2017) in the process of glycolysis. Another rate-controlling enzyme is catalyzed by phosphofructokinase-1 (PFK-1), which regulates the transformation of fructose-6-phosphate (F-6-P) to fructose-1,6-diphosphate (F-1,6-BP), the second step in glycolysis. The activity of PFK-1 is a key factor in the regulation of glycolysis flux. Fructose-2,6-biphosphate (F-2,6-BP), another product of F-6-P in the situation of starvation, is the most effective allosteric activator of PFK-1, which may take effect even in a physiological concentration (De Bock et al., 2013a). Phosphofructokinase-2/fructose-2,6-bisphosphotase (PFKFB) is a bifunctional enzyme that catalyzes the synthesis and hydrolysis of F-2,6-BP. F-1.6-BP then cleaves into dihydroxyacetone phosphate and glyceraldehyde-3-phosphate and produces phosphoenolpyruvic through a series of reactions. Under the catalysis of pyruvate kinase (PK), the high-energy phosphate on phosphoenolpyruvate is transferred to ADP to generate ATP and pyruvate. PK is the other glycolysis rate-controlling enzyme, which converts phosphoenolpyruvate (PEP) to pyruvate. There are four different tissue-specific subtypes of PK. In healthy adults, tumor cells, and embryonic cells, the M2 isoform (PKM2) exists as a dimer or tetramer (Israelsen et al., 2013; Gomez-Escudero et al., 2019). Tetrameric PKM2 has a high affinity for PEP, which promotes glycolysis to produce ATP. While dimeric PKM2 has a low affinity for PEP, which reduces the conversion of pyruvate, thus inducing glycolytic intermediates to switch into the glycolysis side pathway (see Figure 1; Israelsen et al., 2013). A part of pyruvate, the product of glycolysis, enters the tricarboxylic acid cycle, and the other part produces lactate in another way.

The intermediates of glycolysis can be shunted into the side pathway of glycolysis to produce macromolecules and nicotinamide adenine dinucleotide phosphate (NADPH) to maintain redox homeostasis (DeBerardinis et al., 2008; Fitzgerald et al., 2018). The pentose phosphate pathway (PPP) is a side pathway of glycolysis. It refers to the formation of a bypass from G-6-P, an intermediate product of glycolysis. Fructose-6-phosphate (F-6-P) and glyceraldehyde-3-phosphate are produced in two stages; i.e., oxidation and group transfer, thus returning to the glycolysis pathway. PPP does not produce ATP, which in turn produces ribose-5-phosphate (R-5-P) and NADPH for nucleotide synthesis and redox balance (Jongkind et al., 1989). In addition, NADPH is also a hydrogen donor for many anabolisms, participating in hydroxylation and maintaining the reduced state of glutathione (Ghesquiere et al., 2014). Glucose-6-phosphate dehydrogenase (G-6-PD) is the key enzyme of PPP. The activity of G-6-PD determines the flux of glucose-6-phosphate that enters the side pathway (see Figure 1). Inhibition of G-6-PD results in the death of ECs (Vizan et al., 2009), while the overexpression of G-6-PD stimulates endothelial proliferation, migration, and then angiogenesis (Lorenzi, 2007). Moreover, knockout of the G-6-PD gene in embryos leads to early death (Pandolfi et al., 1995).

The hexosamine biosynthesis pathway (HBP) is another side pathway of glycolysis and it is related to post-translational modification and has a low utilization rate in the glucose metabolic capacity. Glutamine fructose-6-phosphate aminotransferase (GFAT) is a rate-controlling enzyme of HBP. The exact role of HBP in the function of ECs is still elusive. It is speculated that the modification of proteins by glycosylation and synthesis of glycolipids, proteoglycans, and glycosylphosphatidylinositol anchors may play a regulatory role (Wells et al., 2001). For example, N-glycosylation enhances the stability, membrane expression, and signal activity of VEGF receptor 2 (VEGFR2) (Cruys et al., 2016), while the interaction of the Notch signal-related ligand is affected by O-glycosylation. In addition, in an aortic ring angiogenesis model, the damage of endothelial cell germination is related to high levels of O-linked N-acetylglucosamine (O-GlcNAc) (Clark et al., 2003; Luo et al., 2008). The underlying mechanism is still unclear (see Figure 1; Li et al., 2019a).

## The Metabolites of Glycolysis in Angiogenesis

The main metabolite of glycolysis is lactate, which is an effective stimulus for angiogenesis by acting as a signal molecule through intracrine, autocrine, or paracrine activities (Parra-Bonilla et al., 2010, 2013). The accumulation of lactate induces a response to hypoxia via the hypoxia-inducible factor (HIF), a central regulator of hypoxia-responsive genes in various processes including angiogenesis (Harris, 2002). Lactate competitively blocks prolyl hydroxylase domain protein 2 (PHD2), a protein that can sense the concentration of oxygen, and thereby activates HIF-1 (Hunt et al., 2007; Sonveaux et al., 2012). HIF-1 induces the upgraded expression of VEFG, the concentration gradient of which guides the direction of vessel sprouting.

Lactate can also participate in the oxidative metabolism. Lee et al. (2015) found a hypoxia response process relating to N-myc downstream regulatory gene 3 (NDRG3), which is independent of HIF. NDRG3 is a newly discovered oxygen regulatory protein degraded by the PHD2/VHL dependent pathway under a physiological oxygen concentration. The degrading process can be blocked by binding with accumulated lactate under hypoxia. This stable NDRG3 binds to c-Raf, mediating activation of the RAF-ERK pathway induced by hypoxia, and promoting angiogenesis and cell growth. Inhibition of intracellular lactate synthesis eliminates NDRG3 mediated hypoxia responses (Lee et al., 2015).

In addition to lactate, pyruvate is also a metabolite of glycolysis. Lactate is oxidized to pyruvate through a series of reactions. At the same time, the volume of NAD + and the expression of the protein poly-ADP-ribosylation are reduced, which promote the deposition of collagen (Green and Goldberg, 1964), enhances the production and activity of VEGF in fibroblasts (Trabold et al., 2003) and macrophages, and promotes angiogenesis.

By adding lactate and pyruvate to HUVECs *in vitro*, the measurements of the results with Western blotting showed that pyruvate has a stronger ability to induce HIF than lactate. The activity of HIF-1 mainly depends on the stability of HIF-1a. When HIF proline hydroxylase (PHD) is as active as in oxidized endothelial cells (ECS), HIF-1a is hydroxylated and localized in the proteasome for degradation, resulting in reduction of the activity of HIF-1. The synthesis of PHD requires 2-oxoglutarate as a substrate, which can be inhibited competitively by pyruvate, thus inducing the upregulation of HIF-1 expression (Sonveaux et al., 2012).

## PFKFB3 in Glycolysis

As mentioned above, F-2,6-BP is the most effective allosteric activator of phosphofructokinase 1 (PFK1), which can be regulated by the bifunctional enzyme 6-phosphofructo-2-kinase/fructose-2,6-bisphosphatase (PFKFB) (De Bock et al., 2013b; Figure 1). The PFKFB family has four isoenzymes; i.e., PFKFB 1–4. Despite the co-expression of these four isoenzymes, which have different effects on the regulation of glycolysis, the specific deletion of *pfkfb3* in HUVECs has the most obvious effect on the inhibition of glycolysis (De Bock et al., 2013b; Xu et al., 2014), indicating PFKFB3 plays a key role in regulating glycolysis flux, by altering the F-2,6-BP level. The ubiquitous enzyme is coded by the *pfkfb3* gene, which is located at 10p15-p14 (Calvo et al., 2006; Fleischer et al., 2011).

## The Role of PFKFB3 in ECs and Angiogenesis

**FIGURE 2:**
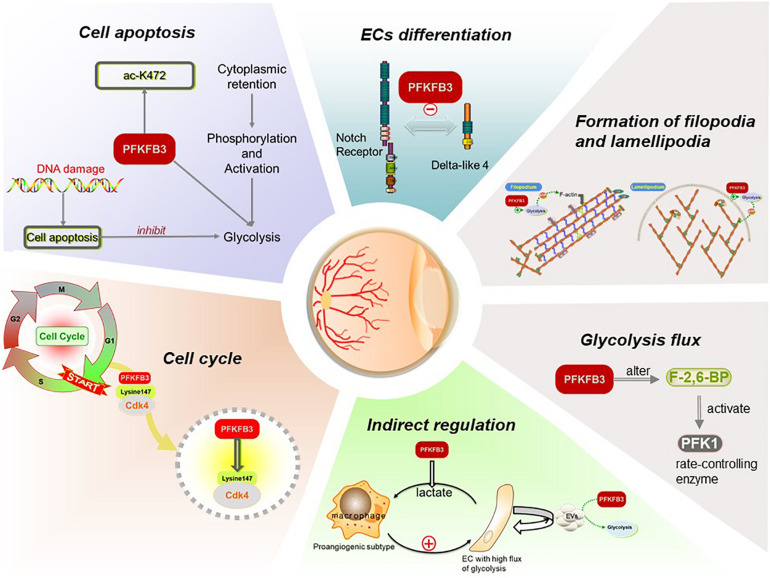
Functions of PFKFB3 plays in ECs in angiogenesis. PFKFB3 promotes the process of angiogenesis by inducing endothelial proliferation and migration. On the one hand, located to the nucleus, PFKFB3 may promote endothelial proliferation by elevating glycolysis flux and regulate cell cycle. On the other, the expression of PFKFB3 regulates the differentiation of ECs, and then promotes the formation of the filopodia and lamellipodia so that the endothelial migration initiates and the vessel sprouts. Besides, the components of extracellular vesicles (EVs) secreted by ECs may be influenced by the overexpression or inhibition of PFKFB3, then regulate the ECs in return.

Many studies have shed light on the significance of the various roles that PFKFB3 plays in angiogenesis, and for the key regulation it exerts on glycolysis (Figure 2; the role PFKFB3 plays in ECs and angiogenesis).

### Elevate Glycolysis Flux

It has been corroborated that PFKFB3 alters the level of F-2,6-BP so that it effectively controls glycolytic flux (De Bock et al., 2013b). Although glycolysis produces less ATP per mole, it can produce the energy ECs need for proliferation faster and promote the uptake of glucose (Heiden et al., 2009; Jones and Thompson, 2009) to achieve more energy production for the sprouting behavior of vessels. Moreover, VEGF may double the flux of glycolysis by upregulating the expression of PFKFB3, even before the ECs begin to migrate as the subtype of tip cells (Sawada and Arany, 2017).

In parallel, downregulation of PFKFB3 may also reduce the production of lactate, which functions as an irritant of angiogenesis (Li et al., 2019a). Lactate uptake by ECs can induce activation of the NF-κB pathway and the expression of IL-8 mediated by ROS (Végran et al., 2011). Also, it regulates the expression of NDRG3, a tumor suppressor protein, which stimulates angiogenesis in a hypoxic environment (Lee et al., 2015).

### Regulation of the Cell Cycle

It has been observed that PFKFB3 localizes to the nucleus whereas other PFKFB enzymes localize to the cytoplasm (Yalcin et al., 2009). PFKFB3 in the nucleus has a positive effect on promotion of the cell cycle. Mass spectrometry analysis has shown that cyclin dependent kinase 4 (CDK4) could control transition of the cell cycle from the G1 phase to S phase, and PFKFB3 interacts with CDK4 in some way. Lysine 147 is the key site for PFKFB3 to bind with CDK4. Binding to PFKFB3 leads to CDK4 protein accumulation by inhibiting ubiquitin proteasome degradation mediated by the heat shock protein 90-cdc37-cdk4 complex. When mutating lysine 147 to alanine, the interactions between PFKFB3 and CDK4 are blocked, and the proteasome dependent degradation of CDK4 is accelerated (Jia et al., 2018). In addition, the accumulation of PFKFB3 in the nucleus increases the expression of cyclin D3 and decreases the expression of p27kip1, thus activating CDK1 to stimulate cellular proliferation (Yalcin et al., 2009; Leal-Esteban and Fajas, 2020). Silencing *pfkfb3* in HUVECs induces the cell cycle to remain in the G0 phase, in which ECs are quiescent and have no possibility of proliferating (De Bock et al., 2013b). In contrast, the growth of ECs with PFKFB3 overexpression increased significantly compared with controls (Xu et al., 2014). These findings suggested that nuclear PFKFB3 signaling plays an important role in regulation of the cell cycle, thus contributing to the status control of ECs.

On the other hand, the ubiquitin proteasome pathway has been known to be a highly selective and important protein degradation pathway in all eukaryotes so far. Proteins that are in the process of being degraded shall first be ubiquitinated and then will be degraded by the proteasome. The ubiquitin proteasome pathway consists of ubiquitin, the ubiquitin activating enzyme E1, the ubiquitin binding enzyme E2, ubiquitin ligase E3, 26S proteasome, and deubiquitinases (DUBs) (Kornitzer and Ciechanover, 2000). Ubiquitin is a small molecular protein with 76 amino acid residues, which is named thus because it is widely distributed in various eukaryotic cells and tissues. Ubiquitin is utilized by cells as a covalent modifier for other proteins to activate their functions and target their degradation, depending on the extent of the binding between ubiquitin and the ubiquitin activating enzyme (Goldstein et al., 1975). APC/C is an evolutionarily conserved E3 ubiquitin ligase. Its main function is to trigger the transition from metaphase to anaphase during mitosis, which plays a key role in cell division, making it crucial for cellular survival and development (Schrock et al., 2020). The ubiquitin ligase APC/C-Cdk-1 can promote ubiquitination and degradation of PFKFB3, thus reducing glycolysis flux, and then generally reducing the number of cells in S phase and inhibiting cell proliferation (Almeida et al., 2010).

### Induction of the Differentiation of ECs

The differentiation of ECs is an imperative part of angiogenesis. As ECs are highly plastic, tip and stalk cells continuously compete for the leading tip position in angiogenesis sprouts (Jakobsson et al., 2010). Although glycolysis flux is high in all types of ECs, it is comparatively low in quiescent ECs because the transcription factors FoxO1 and the Notch signal downregulate the expression of glycolytic genes. It has been reported that glycolysis flux is much higher in tip cells than in other subtypes of ECs (Dumas et al., 2020). The mobility of filopodia depends on the energy supply of ATP during the migration of tip cells. However, due to the large size of mitochondria, they cannot enter the tiny filopodia. The glycolysis pathway can efficiently and rapidly maintain local ATP production, thus promoting the migration and extension of tip cells (Vandekeere et al., 2015).

Growth factors, such as VEGF, upregulate the expression of glycolytic genes in angiogenic endothelial cells, and both proliferative stalk cells and migrating tip cells rely on glycolysis (De Bock et al., 2013b; Yu et al., 2017). In the tip cells, energy is not only produced in the cytoplasm by glycolysis, but also occurs in the local ATP hot spots of filopodia and lamellipodia, which promotes remodeling of the energy intensive actin cytoskeleton and promotes the competitiveness of tip cells (De Bock et al., 2013b).

It has been recognized that the tip-stalk cell selection relies mainly on the Dll4-Notch signaling pathway and the signaling circuit is constantly re-evaluated as the sprouts migrate to different environments (Jakobsson et al., 2010; Moya et al., 2012). Under the condition of hypoxia, VEGF can activate certain ECs, cause differentiation into tip cells, and induce the expression of Dll4 (Lobov and Mikhailova, 2018). Dll4 then activates the Notch signaling pathway in the adjacent stalk cells, thus inhibiting the differentiation of these stalk cells into tip cells (Blanco and Gerhardt, 2013). In addition to Dll4, Jagged1 is also a ligand for vascular ECs. Jagged1 is a weaker activator of Notch signals than Dll4 and is competing with Dll4 to bind to the Notch receptor continuously. It has an antagonistic effect on Dll4-Notch signals (Benedito et al., 2009; Qiu et al., 2016). Intriguingly, lowering glycolytic flux through PFKFB3 silencing compromises both tip cell competitiveness and stalk cell proliferation. Both *in vitro* and *in vivo* studies have revealed that the overexpression of PFKFB3 overrules the negative effects of Notch signaling on angiogenesis, promoting tip cell formation and vessel branching (De Bock et al., 2013b), suggesting that PFKFB3 may play a role in tip-stalk EC selection and the EC metabolism can override genetic tip vs. stalk cues. Notably, the forced induction or repression of PFKFB3 has little influence on the gene expression signature of both tip and stalk cells (De Bock et al., 2013b; Sawada and Arany, 2017). These results have suggested that it is the metabolism pathways, such as glycolysis, that determine differentiation of ECs, rather than genetic signals.

### Promoting the Formation of Filopodia and Lamellipodia

Other studies have reported that when tip cells migrate, the glycolytic enzyme PFKFB3 exists in the filopodia and lamellipodia formed by the enrichment of F-actin, and a large amount of ATP is produced in the folds of the cell membrane enriched with F-actin; at the same time, F-actin enhances the activity of PFKFB3, thus increasing the glycolysis rate, rapidly producing ATP locally, and forming “energy hot spots.” These results may explain why the manipulation of PFKFB3 inhibits the formation of filopodia and lamellipodia in ECs (Vandekeere et al., 2015; Gallop, 2020).

### Suppression of Cell Apoptosis

As reported in a recent study, the 472-lysine residue (k472) of the PFKFB3 protein can be acetylated, which inactivates the nuclear localization signal of PFKFB3 and promotes the retention of PFKFB3 in the cytoplasm (Li et al., 2018). PFKFB3 located in the cytoplasm is more easily phosphorylated by kinase AMPK, leading to the activation of PFKFB3 and promoting glycolysis, thus protecting ECs from apoptosis and promoting the process of angiogenesis.

### Regulating ECs Indirectly

It is also very intriguing that glycolytic metabolites could be initiators of reciprocal activation of macrophages/microglia and ECs. Under highly glycolytic conditions, macrophages/microglia exhibited a unique angiogenic phenotype with increased production of both proinflammatory and proangiogenic cytokines. Knockout of *pfkfb3* in myeloid cells impaired the ability of macrophages/microglia to acquire the angiogenic phenotype, rendering them unable to promote EC proliferation and sprouting and pathological neovascularization in a mouse model of oxygen-induced proliferative retinopathy (Liu et al., 2020).

All the evidence mentioned above is based on results from studies that have already been reported. Other ways that PFKFB3 may regulate ECs have not been completely revealed, but may be demonstrated in the future. For example, the content of extracellular vesicles (EVs) derived from ECs can reflect the state of cells and be used as a diagnostic index of vascular diseases. An increasing number of studies have focused on EVs since many cells communicate through these extracellular signals. EVs contains a variety of components, including metabolites, proteins, and nucleic acids (mRNA, DNA, and miRNA), which may target given cells (Colombo et al., 2014). Especially when the level of blood glucose changes, myocardial cells and myocardial ECs can interact with each other via EVs (Garcia et al., 2016). These EVs can increase the expression of glucose transporters (GLUT1 and GLUT4) in ECs, thus increasing glycolysis flux (Garcia et al., 2016). Therefore, it remains to be determined whether the contents of EVs will change after PFKFB3 is blocked, thus regulating ECs.

## Regulation of PFKFB3

A few angiogenic factors have been implicated to regulate the expression of PFKFB3. Researchers found that VEGF and FGF induced by tip cells stimulate upgrading of the glycolysis level by upregulating the expression of PFKFB3, thus stimulating angiogenesis. In addition, the Notch signal, the evolutionarily conserved pathway in the vascular system, decreases the level of glycolysis by downregulating the expression of PFKFB3 (De Bock et al., 2013b; Li and Carmeliet, 2018). Once neovascularization is blood-perfused, the blood flow impacts the vascular ECs to produce laminar shear stress. Kruppel-like factor 2 (KLF2) is a transcription factor increased by laminar shear stress (Doddaballapur et al., 2015; Wang et al., 2020). KLF2 down-regulates the expression of PFKFB3, thus inhibiting the glycolysis pathway, promoting the transition of ECs to a quiescent state, and then maintaining the barrier function and arranging the vascular flow axis (de Bruin et al., 2016). A recent study by Feng et al. showed the yes-associated protein (YAP), a downstream effector of the Hippo pathway, acts as a transcriptional co-activator working together with transcriptional enhancer activator domain 1 (TEAD1), and binds to the promoter of *pfkfb3*. The YAP1-TEAD1-PFKFB3 axis is crucial for EC glycolysis and consequently influences EC biological functions and ocular angiogenesis (Feng et al., 2021). However, it remains to be seen whether crosstalk exists in these signaling pathways and if yes, the specific mechanisms need to be determined.

It should be noted that the sequence of the *pfkfb3* gene contains multiple serine phosphorylation sites, including s461, S467, and s478. When cells are confronted with hypoxia, high osmotic pressure, and other stress conditions, PFKFB3 will be phosphorylated by activating the p38/MK2, MAPK, ERK/RSK pathways to enhance the activity of PFKFB3 and increase glycolysis flux to meet the energy demands of cell proliferation (Novellasdemunt et al., 2013; Rodriguez-Garcia et al., 2017). MiR-206, miR-26a, miR-26b, and other micro-RNAs can also inhibit the transcriptional activity of PFKFB3 by directly interacting with the 3’-UTR (Ge et al., 2015; Wu et al., 2019), suggesting the possibility of epigenetic regulation of PFKFB3.

## Non-Canonical Roles of PFKFB3

Apart from regulating the process of angiogenesis, manipulating PFKFB3 showed it has a non-canonical role in rebuilding and normalizing the tumor vasculature. Compared to normal ECs, tumor ECs display hyperglycolytic activity and the tumor vasculatures are characterized as being leaky and immature compared to normal vasculatures. PFKFB3-driven glycolysis contributes to the characteristic because the endocytosis of plasma membrane proteins depends on ATP produced by glycolysis. Therefore, the blockade of PFKFB3 enhances plasma membrane exposure to VE-cadherin, resulting in tightening of the barrier (Li et al., 2019b). Similar to ECs, pericytes are also highly glycolytic and require the energy generated by glycolysis to realize the physiological functions. This explains why a PFKFB3 blockade increases pericyte coverage and helps to tighten the barrier as well. Glycolysis induces NF-KB-driven vascular inflammation through lactate signaling, which is why a PFKFB3 blockade reduces vascular inflammation and related cancer cell metastasis (Cantelmo et al., 2016; de Bruin et al., 2016). The inhibition of PFKFB3 may play a positive role in the normalization of tumor vessels so that it helps to reduce tumor metastasis and improve the effect of chemo- and immunotherapy (Li et al., 2019a).

## Novel Targets Found in Endothelial Metabolism Activities

### Manipulating Metabolic Enzymes as Treatment

Manipulating metabolic enzymes in glycolysis offers novel opportunities for treatment. An increasing number of studies have been carried out on the inhibition of PFKFB3. 3-(3-pyridinyl)-1-(4-pyridinyl)-2-propen-1-one (3PO), a small-molecule compound, is a representative inhibitor of PFKFB3 (Clem et al., 2008). It has been reported that the use of 3PO in ECs down-regulates glycolysis flux by 35–40% (Schoors et al., 2014). Inspiringly, the inhibition of PFKFB3 maintained by 3PO is partial and transient, which means once the use of 3PO is stopped, the effects on PFKFB3 inhibition vanish in a short time, leaving few long-term side effects (Perrotta et al., 2020).

Additionally, 3PO inhibits activation of the NF-κB signaling pathway by inhibiting the phosphorylation of p65 and IκBα induced by IL-1β, which cannot be observed with the inhibition of PFKFB3 by YN1 (another inhibitor of PFKFB3). Furthermore, 3PO can also postpone the phosphorylation of IKKα/β and JNK induced by IL-1β and TNF, thus blocking activation of the IKK complex, which is a core part of the NF-κB signaling pathway, and then delaying the progression of inflammation in retinopathy (Wik et al., 2020).

### Manipulating Metabolites as Treatment

Lactate is the major metabolite of glycolysis, which has recently been reported as having a role in angiogenesis, except for upregulating the expression of HIF-1 signals (Harris, 2002). It has been demonstrated that lactate-induced AKT phosphorylation is involved in PFKFB3-driven endothelial angiogenesis (Xu et al., 2014). The lack of PFKFB3 led to a low level of glycolysis and a low level of lactate, which subsequently caused a decrease in pAKT and inhibition of EC proliferation and tube formation. Lactate signals induce vascular inflammation driven by the NF-KB signaling pathway (Li et al., 2019b). Secreted by ECs, the metabolite induces pericytes to produce vasoactive signals, which seems to be regulated in the context of cellular energy homeostasis (Eelen et al., 2018). Lactate arouses vasodilation under the circumstances of a low energy supply, while in the case of a sufficient supply, it arouses vasoconstriction (Eelen et al., 2018; Li et al., 2019a). In addition, lactate regulates gene transcription of immune cells. Lactate plays an essential role in promoting the differentiation of macrophages into the M2 subtype, which has a more anti-inflammatory phenotype, and then the polarized macrophages upregulate the expression of VEGF, which promotes angiogenesis, forming a positive feedback regulatory process, thus stimulating further angiogenesis (Zhang et al., 2020). Lactate enters ECs and functions via monocarboxylate transporters 1 (MCT1) (Murray et al., 2005). The effectiveness of targeting the EC transporter MCT1 in the treatment of ocular neovascular diseases remains to be studied further.

## Ocular Angiogenesis and Glycolysis Inhibition

Recent studies have found that the rate of glycolysis may be a decisive factor in the progression of choroidal neovascularization (CNV). 3PO therapy targeting PFKFB3 reduces injury in CNV and improves the efficiency of anti-vascular therapy by inhibiting VEGFR2 (Schoors et al., 2014; Li et al., 2019a). In a laser-induced CNV in mice, a model of age-related macular degeneration (AMD) (Van de Veire et al., 2010), 3PO reduced the damaged area and increased the anti-angiogenic effect of DC101, a monoclonal antibody of VEGFR2. When the second-best dose of DC101 was used, the area of CNV was reduced by 38%, while the combination of DC101 and 3PO reduced the area of CNV by 67% (Schoors et al., 2014).

In an oxygen-induced retinopathy (OIR) model, treatment with 3PO in ROP mice during the proliferative period from postnatal day 12 (P12) to P17 showed great effects since the formation of vascular clusters in P17 was reduced (Schoors et al., 2014; Rivera et al., 2017). This suggested that 3PO reduced the formation of pathological vessel clusters in the retina in the preclinical ROP model.

These results suggested that blocking PFKFB3 can block the process of pathological angiogenesis to a certain extent (Schoors et al., 2014). Inhibition of PFKFB3 can impede tip cell behavior (Krueger et al., 2011) by disturbing the formation of filopodia as well as the proliferation of endothelial cells, thus inhibiting the sprouting and branching of blood vessels. In addition, it can enhance the effect of a VEGFR blocker, thus producing a better antiangiogenic effect (Schoors et al., 2014). Moreover, the finding that 3PO impairs lumen formation, which is a process requiring actin cytoskeleton changes (Sacharidou et al., 2012), agrees with the finding that PFKFB3 regulates the process of actin cytoskeleton remodeling (De Bock et al., 2013b). Although studies have shown that 3PO can only partially and temporarily reduce glycolysis levels *in vivo*, it suffices to reduce the number of pathological neovascularizations in eyes and inflammatory models (Schoors et al., 2014; Perrotta et al., 2020).

## Glycolysis Inhibition in Other Diseases

### Tumors

The pathological angiogenesis of tumors is induced by a variety of angiogenic factors, including VEGF, FGF, and angiopoietin (Carmeliet and Jain, 2011; Jain, 2014). A hypoxic environment in tumors leads to the activation of HIF-1α, which triggers the expression of various growth factors that promote tumor angiogenesis (Ramjiawan et al., 2017). Compared with normal endothelial cells (NECs), tumor endothelial cells (TECs) are characterized by higher glycolysis flux and a higher proliferation rate. In tumor-bearing mice, specific deletion of a single allele of the *pfkfb3* gene in ECs reduced the flux of glycolysis by 15–20% (Cantelmo et al., 2016), which normalizes the tumor vessels though a series of regulating processes. As endocytosis in endothelial cells requires ATP from glycolysis, inhibition of PFKFB3 promotes high-level expression of VE-cadherin in ECs, which tightens the vascular barrier and reduces the invasion of cancer cells. Therefore, targeting glycolysis of TECs may provide a novel therapeutic strategy for reducing tumor metastasis, normalizing tumor blood vessels, and improving chemo-and immunotherapy (Ramjiawan et al., 2017; Li et al., 2019a).

### Pulmonary Arterial Hypertension

Pulmonary arterial hypertension (PAH) has also been described as a vasculopathy, which is often fatal. The pathophysiology of PAH includes pathological angiogenesis, extensive vascular remodeling and pulmonary vascular lesions (Farber and Loscalzo, 2004; Leopold and Maron, 2016; Dodson et al., 2018). In PAH, glucose metabolism switches to glycolysis, and PFKFB3 plays an important role in the proliferation and collagen synthesis of pulmonary artery smooth muscle cells (PASMCs). It has been shown that increased expression of PFKFB3 and a high level of lactate may induce ERK1/2 phosphorylation and calpain activation in PAH. Inhibition of PFKFB3 attenuates the activation of calpain, collagen synthesis, and proliferation of PASMCs induced by PDGF. More intriguingly, specific-SMC knockout of the PFKFB3 inhibitor attenuates the progression of PAH and vascular remodeling in animal PAH models. These findings suggest that PFKFB3 may be a new target in the treatment of PAH (Kovacs et al., 2019).

### Atherosclerosis

Atherosclerosis is also a vascular disease with high morbidity and mortality. Intraplaque neovascularization is associated with atherosclerosis progression (Carmeliet, 2003; Virmani et al., 2005; Van der Veken et al., 2016). Partial inhibition of glycolysis reduces intraplaque neovascularization, while few changes in the composition of the plaque can be observed. It has been reported that 3PO inhibits the formation of coronary plaque and improves cardiac functions. Although 3PO is an inhibitor of PFKFB3, a key enzyme in the glycolysis pathway, recent studies have reported that 3PO may not bind to PFKFB3, indicating the functions of 3PO may be unrelated to PFKFB3 in some way (Veseli et al., 2020). Whether PFKFB3 is a potential target for the treatment of atherosclerotic plaque with 3PO remains to be corroborated. In addition, downregulation of endothelial adhesion molecule expression by 3PO mainly affects the initial stage of the development of atherosclerosis (Perrotta et al., 2020). Therefore, it is speculated that preventive use of 3PO in the early stage of plaque formation can improve cardiac function.

## Conclusion and Perspectives

Endothelial cell metabolism is regulated by a series of complicated mechanisms and it plays an important role in angiogenesis. Angiogenic ECs have a great demand for energy, which is different from quiescent ECs, and this energy is vital for the initiation of cellular proliferation and migration (Potente and Carmeliet, 2017). Glycolysis provides most of the ATP that is required by ECs, which makes its key regulating enzyme PFKFB3 a principal target in studies on endothelial cell metabolism. At the same time, only 2.5% of ATP in the human body is produced by glycolysis. Therefore, inhibiting the process of glycolysis may have fewer side effects than other therapies that have been applied (Bousseau et al., 2018).

Despite numerous studies on PFKFB3, the underlying mechanisms of how PFKFB3 is regulated is still elusive. On the one hand, there are few reports on inhibitors of PFKFB3, whose functions and mechanisms remain to be fully elucidated. On the other hand, the safety of therapies that inhibit PFKFB3 is questionable in certain circumstances. As a report indicated, the use of 3PO in high concentrations may cause tumor hypoxia and weakening of the vascular barrier integrity, which leads to tumor vessels becoming leaky, as well as the increasing risk of tumor metastasis (Conradi et al., 2017). Also, how the Notch signaling pathway regulates PFKFB3 and the crosstalk between PFKFB3 and the VEGF pathway are still unknown. However, as there is a crucial role for PFKFB3 in angiogenesis, the use of a PFKFB3 inhibitor in the treatment of ocular neovascular diseases has been shown to have potential. After additional research on the crosstalk between these pathways, we may have more feasible targets for clinical treatment. In parallel, considering the plasticity of ECs, the technique of single cell sequencing will be a powerful tool for further exploration in this field.

In conclusion, the studies on endothelial glycolysis have provided novel targets in the treatment of ocular neovascular diseases. Since countless patients are suffering from blindness that arise from ocular pathological angiogenesis, novel treatment modalities are necessary. With PFK158 as a first-in-human and first-in-class PFKFB3 inhibitor that has been included in a phase I clinical trial (NCT02044861) for the treatment of cancer (Clem et al., 2016), we believe that the clinical application of drugs that target endothelial glycolysis in ocular diseases will be a likely development in the future.

## Author Contributions

Z-YZ and G-RD produced idea of this review. Z-YZ, LW, and G-RD performed literature search and data analysis. G-RD and Y-SW critically revised the work. All authors contributed to the study conception and design, and read and approved the final manuscript.

## Conflict of Interest

The authors declare that the research was conducted in the absence of any commercial or financial relationships that could be construed as a potential conflict of interest.
